# *Iodobacter fluviatilis*, a New Potential Opportunistic Pathogen Associated with Skin Lesions, First Report in *Hypophthalmichthys nobilis* in China

**DOI:** 10.3390/pathogens14100978

**Published:** 2025-09-26

**Authors:** Kai Chen, Nannan Shen, Ting Qin, Liushen Lu, Dongpo Xu, Bingwen Xi, Jun Xie

**Affiliations:** 1 Key Laboratory of Aquatic Animal Nutrition and Health, Freshwater Fisheries Research Center, Chinese Academy of Fishery Science, Wuxi 214081, China; chenk@ffrc.cn (K.C.); qint@ffrc.cn (T.Q.); luls@ffrc.cn (L.L.); xudp@ffrc.cn (D.X.); 2 Wuxi Fishery College, Nanjing Agricultural University, Wuxi 214081, China; 2023113017@stu.njau.edu.cn

**Keywords:** *Iodobacter fluviatilis*, isolation, identification, drug susceptibility, histopathology, *Hypophthalmichthys nobilis*

## Abstract

In the spring of 2023, a disease outbreak occurred in Lake Taihu in China, which caused a large number of deaths of *H. nobilis*. In order to investigate the cause of morbidity and mortality of the *H. nobilis*, the diseased fish were collected for histopathological and etiological studies. Histopathological observation revealed that substantial inflammatory cell infiltration was observed around skin lesion in diseased fish, extensive degeneration and necrosis were observed in the hepatic parenchymal cells, the spleen exhibited congestion, and the kidney showed congestion. A bacterial strain, C1, isolated from diseased *H. nobilis* was identified as *Iodobacter fluviatilis* through *16S rRNA* gene sequencing and biochemical phenotypic characterization. Experimental infection of the fish via intramuscular injection induced a localized abscess in a subset of fish. Antimicrobial susceptibility testing revealed that the isolate was susceptible to aminoglycosides, tetracyclines, quinolones and amphenicols, but resistant to sulfonamides commonly used in aquaculture. Here, we describe an association between *I. fluviatilis* and skin lesions in *H. nobilis*. Furthermore, we report the biochemical characteristics and drug resistance profile of the isolated bacteria. These findings also facilitate further investigations into the role of *I. fluviatilis* associated with skin diseases of *H. nobilis* and other freshwater fish.

## 1. Introduction

*Hypophthalmichthys nobilis*, bighead carp, is one of the most economically important fresh-water farmed fish species in China, with an annual production exceeding 3 million tons in recent years [1]. As a phytoplankton-feeding species, *H. nobilis* plays a key role in traditional polyculture systems by helping mitigate eutrophication [2]. Widespread eutrophication in shallow lakes [3] and efforts to control harmful algal blooms (HABs) using zooplankton have often been unsuccessful [4]. In contrast, *H. nobilis* has replaced zooplankton as a more effective biological control agent [5,6]. As a result, it has been widely introduced into eutrophic lakes to suppress cyanobacterial blooms [7,8], leading to its establishment as a dominant species in many lakes and reservoirs across China.

Current studies indicate that pathogens affecting *H. nobilis* (e.g., parasites and bacteria) pose significant health risks [9]. Among these pathogens, the most prevalent parasites include *Sinergasilus polycolpus* [10,11,12], *Myxobolus pavlovskii* [13] and the newly discovered *Limnotrachelobdella hypophthalmichthysa* [14]. Bacterial infections are predominantly associated with *Aeromonas* sp., which is the primary causative agent of diseases in *H. nobilis*. The pathogenicity of *Aeromonas punctata* [15], *Aeromonas hydrophila* subsp. *hydrophila* [16], *Aeromonas caviae* [17] and *Aeromonas veronii* [18] in *H. nobilis* has been successively demonstrated in multiple studies. Furthermore, research has revealed that *Pseudomonas putide* [19], *Yersinia ruckeri* [20,21] and *Streptococcus agalactiae* [22] exhibits pathogenic potential in this species. These pathogenic bacteria induce characteristic clinical signs, including cutaneous hemorrhagic lesions, localized inflammation, and mortality in severe cases.

In February 2023, a disease outbreak occurred in Lake Taihu in China, resulting in significant mortality among *H. nobilis*. A suspected bacterial strain C1 was isolated from diseased *H. nobilis* and identified through morphological, biochemical, and *16S rRNA* gene sequence analyses. Histopathological examination of diseased fish and antimicrobial susceptibility testing of the bacterial isolate were conducted. Our study identifies *I. fluviatilis* as a potential opportunistic pathogen posing a risk to *H. nobilis* and other freshwater fish, particularly in connection with skin lesions.

## 2. Materials and Methods

### 2.1. Clinical Signs and Sample Collection

Fish samples were examined by gross visual observation to grasp the clinical signs and macroscopic parasites initially. Then wet smears of diseased fish were prepared for further laboratory diagnosis with a microscope (Leica DM1000, Hesse, Germany). In addition, the liver, spleen, kidney and skin of diseased *H. nobilis* (euthanized with 100 μg/mL MS-222 buffered with sodium bicarbonate) were collected, fixed with 10% formalin. Then, the preserved tissues were routinely dehydrated then embedded in paraffin. Finally, tissue sections (5 µm thickness) were prepared and stained with H&E (Jiancheng, Nanjing, China) for histopathological observation.

### 2.2. Bacterial Isolation and Characteristics

The body surface of diseased *H. nobilis* was disinfected. According to Chen’s description [23], bacteria were isolated from the edge of subcutaneous lesions with sterile inoculating loop, streaked on nutrient agar plates (NA; HopeBio, Qingdao, China) with the quadrant streaking method, and then incubated at 15 °C for 48 h. The suspected dominant single colony type was inoculated into nutrient broth (NB; HopeBio, Qingdao, China) liquid medium and cultured with shaking at 180 rpm at 15 °C to obtain pure culture.

Several smears of the pure culture were prepared by the way described by Chen [23], then stained with Gram-stain Kit according to the manufacturer’s instructions (Jiancheng, Nanjing, China). Finally, the slides were examined and photographed under the light microscope (Leica DM1000, Leica MC170 HD, Hesse, Germany).

To characterize the isolate strain abilities in carbohydrate metabolism, amino acids/protein degradation and carbon source utilization, the culture in the logarithmic growth period were adjusted to 1 × 10^5^ CFU/mL, then inoculated 50 μL suspension into the commercial test tubes at 15 °C for 48 h. The results were interpreted based on the user manual.

At the same time, catalase, oxidase and motility tests were carried out with hydrogen peroxide, p-phenylenediamine oxidation and semi-soft medium methods as described by Mahon [24].

### 2.3. Phylogenetic Analysis of 16S RNA

As described by Chen [23], the nucleic acids of the isolate strain were prepared as the PCR template with the UNIQ-10 column bacterial genome extraction kit (Sangon Biotech, Shanghai, China), then mixed with the universal primers 27F (5′-AGAGTTTGATCATGGCTCAG-3′), 1492R (5′-TACGGTTACCTTGTTACGACTT-3′) and the 2× Taq Plus Mast Mix II (Vazyme Biotech, Nanjing, China) to amplify the 16S rRNA gene following the amplification program (95 °C for 5 min; 35 cycles of 95 °C for 30 s, 60 °C for 30 s, and 72 °C for 30 s; and 72 °C for 5 min) in a T100^TM^ Thermal Cycler (Bio-Rad, Hercules, CA, USA). The amplification products were sent to Sangon Biotech (Shanghai, China) for sequencing. Then, a comparative analysis of the C1 isolate was undertaken using the NCBI (http://blast.ncbi.nlm.nih.gov (accessed on 4 March 2023)). Finally, a phylogenetic tree, containing other 10 strain sequences obtained from the GenBank database of nucleic acid sequences which showed high similarity to isolate strain *16S rRNA*, was constructed with the neighbor-joining method in MEGA-X and tested by bootstrap (1000 repetitions) [25].

### 2.4. Pathogenicity Assay

135 Gibel carp (*Carassius gibelio*), from the experimental station of Freshwater Fisheries Research Center, were used for pathogenicity assay in laboratory. The fish (25–30 g) was arbitrarily assigned into 3 experimental groups (control group, intraperitoneal injection group and intramuscular injection group). Three parallels were set for each group (45 fish).

Based on the different groupings, fish was injected with 100 μL the isolate bacterial cells suspension (3 × 10^7^ CFU/mL) or a substitute, sterilized saline, after anaesthetizing with MS-222 buffer solution (50 μg/mL), respectively. After that, fish was returned to the corresponding aquariums, respectively, and monitored for 15 days. During the observation period, a 1% commercial feed was given to different groups twice per day according to body weight. At the end, the individuals with disease symptoms were used to re-isolate and identify the pathogenic bacteria again with MS-222 buffer solution euthanized (100 μg/mL).

In addition, the tissue surrounding the lesion from natural and artificial infected individuals were collected, then sent to Sangon Biotech (China) for *16S rRNA* gene high-throughput sequencing using the universal primers Nobar_341F: 5′-CCTACGGGNGGCWGCAG-3′, Nobar_805R: 5′-GACTACHVGGGTATCTAATCC-3′. The sequencing results were used for comparison and analysis the characteristics of bacterial communities.

This study was carried out in strict accordance with the recommendations in the Guide for the Care and Use of Laboratory Animals (http://www.nap.edu/catalog/12910.html (accessed on 7 March 2023)). Before the experiment, all fish was acclimatized in indoor water recirculation culture system at 15 ± 1 °C and fed with the commercial feed twice a day. The protocol was approved by the Committee on the Ethics of Animal Experiments of the Freshwater Fisheries Research Center (Authorization Number: 20230313002). All experimental procedures were performed under MS-222 buffer solution, and all efforts were made to minimize suffering.

### 2.5. Drug Sensitivity Tests

The disc diffusion method described by Chen [23] was used for drug susceptibility testing with the following steps. First, the NA plates surface spread with C1 bacterial cells (1 × 10^7^ CFU/mL, 200 µL per plate) were prepared. Then the paper disks with antimicrobial agents were placed on the surface of NA medium. After that, the diameter of the inhibition zones was measured after incubating at 15 °C for 48 h. Finally, the susceptible, intermediate, or resistant criteria for each antimicrobial agent were qualitatively interpreted according to the criteria described by Chen [23].

## 3. Results

### 3.1. Clinical and Histopathological Changes

**Figure 1:**
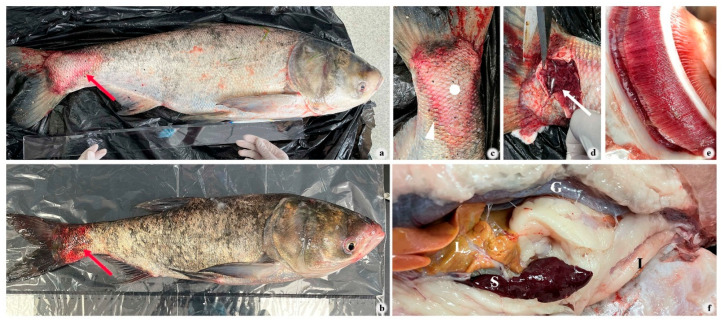
Clinical symptoms of diseased *H. nobilis.* (**a**,**b**): the overall appearance of disease fish, typical local lesion (red arrows); (**c**): caudal peduncle, localized abscess (white spot), localized hemorrhagic inflammation (white triangle); (**d**): subcutaneous edema (white arrows); (**e**): gills; (**f**): internal organs; I, intestine; S, spleen; L, liver; G, gonad.

The disease outbreak occurred in *H. nobilis* in Lake Taihu at a water temperature of approximately 15 °C. Affected fish exhibited clinical signs, including skin pallor with petechial hemorrhages (Figure 1a), cephalic and fin base hemorrhages (Figure 1b), edema (Figure 1c,d), and pale gills (Figure 1e). Gross internal examination identified several abnormalities: marked splenomegaly, a liver presenting with a yellowish discoloration (Figure 1f) and a complete absence of food in the intestine (Figure 1f).

**Figure 2:**
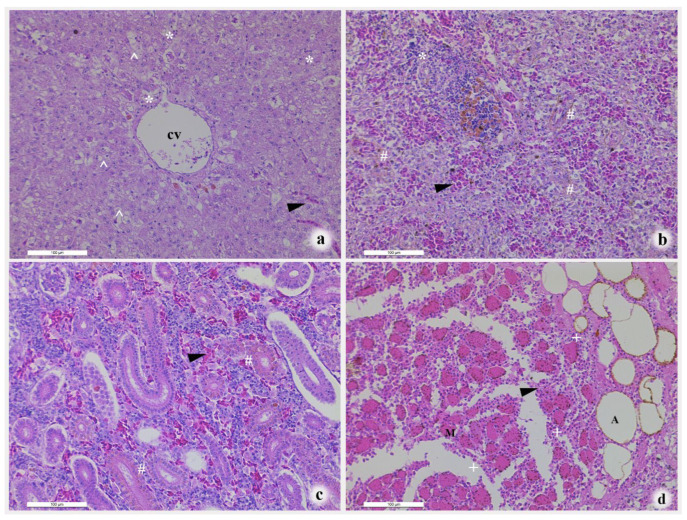
Histopathological changes in diseased *H. nobilis*. (**a**) liver, (**b**) spleen, (**c**) kidney, (**d**) local lesion. Bars = 100 μm; black arrow, red blood cells; “^”, cell membrane rupture; “#”, congestion; “*”, lymphocytes aggregates; CV, central veins; M, muscle fibers; A, adipose cells; “+”, pleomorphic inflammatory cell.

Histopathological analysis of the liver revealed degenerative and inflammatory changes (Figure 2a). Hepatocytes exhibited cytoplasmic vacuolation and marked swelling, with some cells undergoing membrane rupture due to extreme distension. Mild lymphocyte infiltration was observed around central veins, necrotic foci, and sinusoids.

Splenic lesions included congestion of sinuses with erythrocyte accumulation (black arrow) (Figure 2b). Lymphocyte aggregates were frequently observed in the white pulp.

A key characteristic of the renal pathology was peritubular capillary congestion, manifested by accumulations of erythrocytes (Figure 2c).

Upon histopathological examination, lesions penetrating the muscularis were associated with a disruption of vascular integrity and subsequent hemorrhage (Figure 2d) evidenced by extravasated erythrocytes and a pronounced pleomorphic inflammatory cell infiltrate within the spaces between muscle fibers.

### 3.2. Pathogen Isolation and Morphological Observation

**Figure 3:**
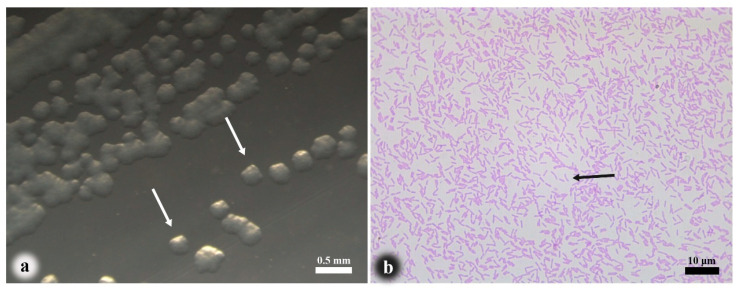
Morphological characteristics of isolate strain C1. (**a**) Colonies, (**b**) Bacterial cells. Colonies on nutrient agar (white arrows); Gram-negative short rod-shaped bacteria (black arrows).

The dominant bacterial strain, C1, was isolated from diseased fish. Colonies (≈0.3 mm in diameter) formed after 48-h incubation at 15 °C on NA medium, exhibiting translucent, circular morphology with entire margins and smooth surfaces (Figure 3a). Gram staining revealed Gram-negative bacilli, and microscopic examination showed rod-shaped bacteria with straight sides and rounded ends (Figure 3b).

### 3.3. Biochemical Identification of Bacteria

The isolate was identified as an oxidase-positive, facultatively anaerobic Gram-negative bacillus through 43 physiological and biochemical tests. Detailed physiological and biochemical characteristics are presented in Table 1.

### 3.4. Bacterial 16S RNA Gene Sequence Analysis

**Figure 4:**
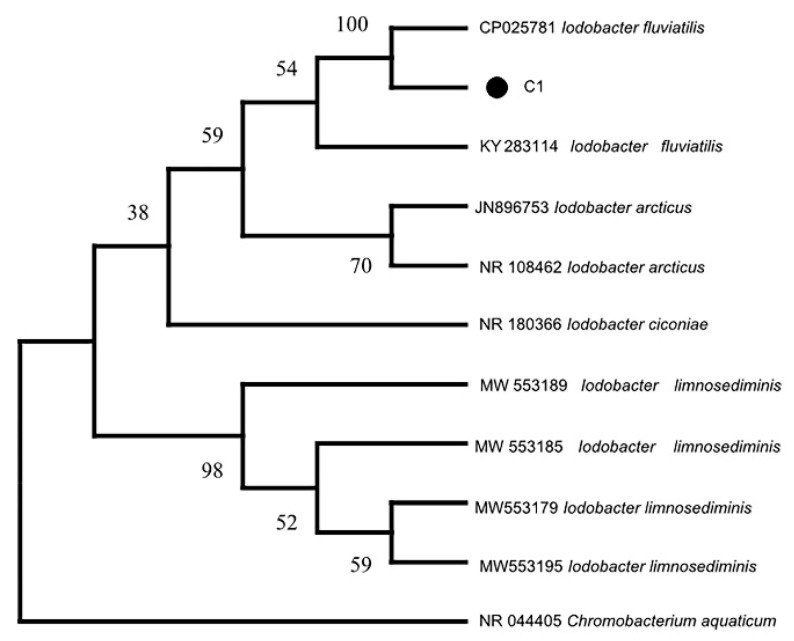
Phylogenetic tree based on the partial *16S rRNA* gene sequences.

A 1420 bp *16S rRNA* gene fragment was amplified from strain C1 using primers 27 F/1492 R. Basic bioinformatic analysis was performed using NCBI BLAST + 2.13.0. The *16S rRNA* sequence of the strain showed 99% query coverage and 99.65% identity to *I. fluviatilis*. Furthermore, phylogenetic analysis included sequences from representative Iodobacter species (*I. fluviatilis*, *I. limnosediminis*, *I. ciconiae* and *I. arcticus*) and *Chromobacterium aquaticum* as an outgroup. The phylogenetic tree constructed using the neighbor-joining method in MEGA X showed that Strain C1 clusters with *I. fluviatilis* (GenBank accession number: CP025781, KY283114), forming a distinct clade (Figure 4). Based on this result and the biochemical characteristics described in Section 3.3, Strain C1 was identified as *I. fluviatilis*.

### 3.5. Pathogenicity

**Figure 5:**
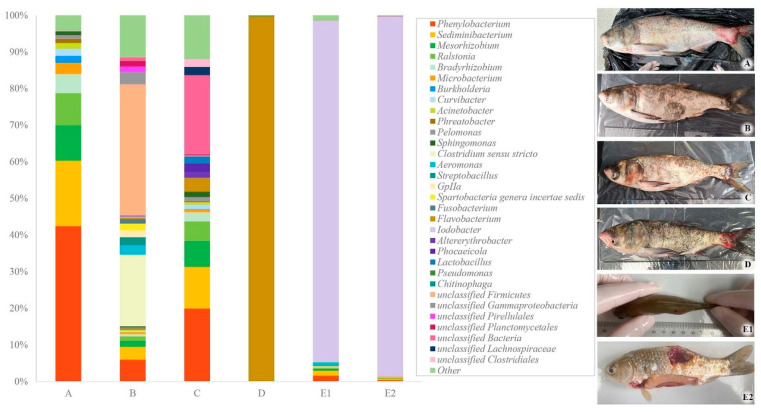
Bacterial communities at the lesion site and major clinical signs in experimental fish. (**A**–**D**) naturally infected fish, (**E1**,**E2**) experiment fish.

Although no mortality occurred in either the experimental or control groups throughout the experiment, some fish (13.3%) in the intramuscular injection group exhibited persistent localized abscess, including hemorrhage and swelling near the injection site (Figure 5(E1,E2)). The pathogen was reisolated from lesioned fish and identified as *I. fluviatilis*, whereas no bacteria were isolated from the intraperitoneal injection group.

*16S rRNA* gene high-throughput sequencing revealed significant variation in bacterial diversity at lesion sites among naturally infected fish (Figure 5A–D), with most exhibiting greater diversity (Shannon index, 2.76) than experimentally infected individuals (Shannon index, 0.27) (Figure 5(E1,E2)). Although *I. fluviatilis* was isolated from naturally infected fish, Iodobacter abundance remained below 0.5%, significantly lower than in experimentally infected fish (>93%) with the localized abscess.

Collectively, these findings suggest that the *I. fluviatilis* strain C1 is an opportunistic pathogen associated with skin lesions under specific conditions.

### 3.6. Drug Sensitivity

Antimicrobial susceptibility testing was performed against 30 antibiotics, revealing that the strain C1 was sensitive to 20 agents, moderately sensitive to 3, and resistant to 7. Detailed susceptibility data are presented in Table 2.

## 4. Discussion

Bacteria are ubiquitous in freshwater aquaculture environments, forming extensive populations in a wide range of habitats including ponds, lakes, and rivers [31]. The composition of fish-colonized bacterial communities is strongly influenced by aquatic environment, yet distinguishing commensal and pathogenic members of the fish microbiota remains challenging [32]. In early research, many bacteria commonly found in the fish microflora have, at one time or another, been associated with fish diseases [33]. However, as research progresses, it has become clear that not all these bacteria are primary pathogens [34]. Many are opportunistic pathogens that only infect Immunosuppressed [35,36], injured [37,38], or physiologically stressed [39] fish. With increasing water contamination and the expansion of intensive aquaculture, bacterial diseases have become more prevalent, causing significant economic losses.

The *I. fluviatilis* studied here was first described in 1989 after isolation from running freshwaters in the UK [27]. Subsequent studies identified three species within the genus *Iodobacter*: *Iodobacter arcticus* [26], *Iodobacter limnosediminis* [29] and *Iodobacter ciconiae* [28]. Prior to 2021 [32], despite being not “new” bacterium, *Iodobacter* strains were rarely reported as causative agents of diseases in aquatic animals. Until recently, *Iodobacter* sp. was not recognized as a fish pathogen, but an investigation has now linked *I. limnosediminis* [32] to skin lesions in freshwater fish. Similarly to Korkea-Aho’s findings on *I. limnosediminis* [32], this study confirms its association with skin lesions. Pathogenicity assays confirmed the causal relationship: intramuscular injection of *I. fluviatilis* induced skin lesions in healthy fish, and the bacterium was reisolated from lesioned tissues.

This study indicates that *I. fluviatilis* strain C1 is an opportunistic pathogen capable of causing skin lesions under specific conditions. Immunosuppression induced by low temperatures and prolonged starvation likely contributes to infections by *I. fluviatilis*, which is typically benign in warm, nutrient-rich conditions but becomes pathogenic in winter [40]. Disease in fish is closely linked to environmental stress [41]. Human activities have significantly impacted aquatic environments, leading to numerous challenges. Pollutants such as plastic [42], hydrocarbons [35], heavy metals [43], and pesticides [44] have damaged freshwater ecosystems. These pollutants rapidly enter aquatic systems, compromising the health of aquatic animals and leading to immunosuppression and damage to immunological barriers [36]. A weakened immune system increases susceptibility to opportunistic pathogens, such as *I. limnosediminis* [32], *Flavobacterium johnsoniae* [37], *Vibrio harveyi* [38], *Aeromonas* [45,46], and even normal microbiota. Furthermore, compared to wild fish, captive fish in extensive and intensive systems face greater environmental stress and have less possibility to escape a negative environment [47]. This increases their risk of opportunistic infections. Therefore, whether in natural waters, fishery facility or pond culture, opportunistic pathogens such as *I. fluviatilis* require greater attention in the face of deteriorating aquaculture environments.

Initially, we aimed to utilize morphological, staining, and biochemical characteristics for auxiliary taxonomic identification. However, limited literature [26,27,28,29] is available on this species, particularly regarding its biochemical phenotypes. Therefore, identification in this study primarily relied on *16S rRNA* gene sequence analysis. Understanding the phenotypic characteristics of opportunistic pathogens can reveal their environmental adaptability [48,49] and contribute to the prevention and treatment of fish diseases, particularly through the characterization of drug resistance. While Gram staining is sufficient to guide initial therapy in some cases, antibiotic susceptibility testing provides better guidance for precise drug application amid the current antibiotic resistance crisis [50]. Furthermore, bacterial phenotypes are valuable not only for identification and antimicrobial application [51,52] but also for indirectly assessing the virulence [53,54] of clinical strains. These phenotypic data will contribute to controlling clinical infections and developing anti-virulence strategies in the future.

In challenge experiments, intramuscular injection of *I. fluviatilis* induced persistent localized abscess similar to those observed in naturally diseased fish. This suggests that *I. fluviatilis* C1 is potentially pathogenic. However, Iodobacter abundance in naturally diseased fish remained below 0.5%. The criterion of Koch’s postulates is not applicable here, because opportunistic pathogens can colonize healthy hosts asymptomatically and may not dominate disease lesions [55].

Host immunity plays a decisive role in disease prognosis, as opportunistic pathogens primarily infect immunocompromised individuals [56]. In the injection challenge model, *I. fluviatilis* avoids external defenses, leading to three possible outcomes: clearance, persistent infection, or mortality. Two outcomes were observed: (1) clearance, characterized by rapid elimination of *I. fluviatilis* without lesions or bacterial recovery (intraperitoneal injection group), and (2) persistent infection, which reflected a stalemate between the pathogen and the immune system, leading to a persistent localized abscess and *I. fluviatilis* persistence (intramuscular injection group). Additionally, due to variability in immune function among healthy individuals, only 13.3% of experimental fish developed disease.

Furthermore, healthy individuals typically have robust immune function. Intramuscular injection bypasses the skin barrier with minimal damage, leaving external defenses functional even in diseased fish throughout the experiment. This prevents invasion by other pathogens [57], resulting in a near-monoculture of Iodobacter (>93%) in experimentally infected fish with the localized abscess. In contrast, naturally diseased fish often exhibit immune dysfunction, and compromised skin barriers allow environmental bacteria to invade, leading to polymicrobial infections including *I. fluviatilis*. The low Iodobacter abundance (<5%) in naturally diseased fish likely reflects the composition of their surface microbiota [32]. These results suggest that opportunistic infections are driven by both host immunocompromise and stochastic interactions between environmental/host microbiota and pathogen populations. From an etiological perspective, greater attention must be given to opportunistic pathogens.

## 5. Conclusions

Here, we describe an association between *I. fluviatilis* and skin lesions in *H. nobilis* and report the biochemical characteristics and drug resistance profile of the isolated bacteria. With increasing water contamination and the expansion of intensive aquaculture, greater attention must be given to opportunistic pathogens such as *I. fluviatilis*.

It is important to clarify that, due to inherent limitations in sample collection, we were unable to obtain a larger dataset. As filter-feeding ectothermic animals, it is an objective fact that the fish experienced prolonged periods of low temperature and food scarcity. However, the potential roles of additional stressors—such as winter stress, pollution, or immunosuppression—require further support through water quality monitoring and host physiological data.

Furthermore, although we took into account both the host specificity of the bacterial pathogen and the natural co-distribution of the two fish species in the affected area, the use of *C. gibelio* as a substitute for pathogenicity testing may still be subject to debate.

## Figures and Tables

**Table 1:** Biochemical characteristics of *Iodobacter fluviatilis* strains and isolates.

Characters	Strains/Isolates						Characters	Strains/Isolates					
	C1	1	2	3	4	5		C1	1	2	3	4	5
Trehalose	+	+	+	N	N	+	Urea hydrolysis	−	−	N	N	−	N
Glucose	+	+	N	N	+	N	ONPG test	−	−	N	N	−	N
Maltose	+	+	+	+	+	+	Gluconate utilization	−	+	V	N	+	N
Mannitol	−	−	−	N	−	N	Citrate utilization	−	+	N	N	N	N
Sucrose	+	−	N	−	N	N	Malonate utilization	−	+	N	N	N	N
Arabinose	+	−	−	−	−	−	OF Test	F	N	F	N	N	F
Xylose	−	−	N	N	N	N	Growth in 6.5% NaCl	−	N	N	N	−	N
Raffinose	−	−	N	N	N	N	Nitrate reduced	+	+	N	N	+	+
Fructose	−	+	N	+	N	N	Phe deaminase	−	−	N	N	N	N
Galactose	−	−	−	N	N	N	Motility	+	N	N	N	N	N
Sorbose	−	−	N	N	N	N	H_2_S	−	−	N	N	N	N
Cellobiose	−	−	−	N	N	N	Gelatin hydrolysis	−	+	N	N	+	+
Melibiose	−	−	N	+	N	N	Catalase	+	N	N	N	+	N
Melizitose	−	−	N	N	N	N	Oxidase	+	+	N	N	+	N
Inulin	+	−	N	N	N	N	Voges–Proskauer test	−	−	N	N	N	N
Mannose	+	+	N	+	+	N	Methyl red test	+	−	N	N	N	N
Dulcitol	−	−	N	N	N	N	Orn decarboxylase	−	−	N	N	N	N
Inositol	−	−	−	N	N	−	Lys decarboxylase	−	−	N	N	N	N
Sorbitol	−	−	N	N	N	N	Arg decarboxylase	−	−	N	N	N	N
Salicin	−	−	N	N	N	N	Arg dihydrolase	−	N	−	+	−	−
Esculin hydrolysis	−	−	−	N	N	−	Indole	−	−	N	N	−	−
Bile esculin test	−	N	N	N	N	N							

Note: (1) 1, *I. fluviatilis* JCM 9044 [26]; 2, *I. fluviatilis* [27]; 3, *I. fluviatilis* DSM 3764 [28]; 4, *I. fluviatilis* ATCC 33051 [29]; 5, *I. fluviatilis* [30]. (2) +, positive; -, negative; F, fermented; V, variable; N, no records.

**Table 2:** Drug sensitivity test results of the strain C1.

Antibiotic	Disk Potency	Inhibition Zone Diameter (mm)	Result	Antibiotic	Disk Potency	Inhibition Zone Diameter (mm)	Result
PEN	10 U	24	S	MI	30 µg	23	S
OX	10 µg	6	R	E	15 µg	15	I
AMP	10 µg	27	S	AZI	15 µg	18	I
PIP	100 µg	30	S	NOR	10 µg	21	S
CN	30 µg	21	S	CIP	5 µg	24	S
CZ	30 µg	35	S	MY	2 µg	6	R
CXM	30 µg	28	S	VAN	30 µg	6	R
CAZ	30 µg	24	S	PB	300 IU	11	R
CTR	30 µg	25	S	SXT	25 µg	12	R
CPZ	75 µg	25	S	C	30 µg	27	S
AMK	30 µg	22	S	CC	2 µg	6	R
GEN	10 µg	18	I	LEV	5 µg	21	S
KAN	30 µg	20	S	IPM	10 µg	35	S
S	10 µg	14	R	DO	30 µg	21	S
TET	30 µg	22	S	FFC	30 µg	31	S
ENR	10 µg	26	S	N	30 µg	24	S

Note: (1) Report categorical result as either susceptible (S), intermediate (I), or resistant (R). (2) R: Inhibition Zone Diameter < 15 mm; I: Inhibition Zone Diameter ranged from 15 mm to 20 mm; S: Inhibition Zone Diameter > 20 mm. (3) PEN, Penicillin; OX, Oxacillin; AMP, Ampicillin; PIP, Piperacillin; CN, Cefalexin; CZ, Cefazolin; CXM, Cefuroxime; CAZ, Ceftazidime; CTR, Ceftriaxone; CPZ, Cefoperazone; AMK, Amikacin; GEN, Gentamicin; KAN, Kanamycin; S, Streptomycin; TET, Tetracycline; MI, Minocycline; E, Erythromycin; AZI, Azithromycin; NOR, Norfloxacin; CIP, Ciprofloxacin; MY, Lincomycin; VAN, Vancomycin; PB, Polymyxin B; SXT, Co-trimoxazole; C, Chloramphenicol; CC, Clindamycin; LEV, Levofloxacin; IPM, Imipenem; DO, Doxycycline; FFC, Florfenicol; ENR, Enrofloxacin; N, Neomycin.

## Data Availability

No new data were created or analyzed in this study.
